# L-DOPA regulates neuroinflammation and Aβ pathology through NEP and ADAM17 in a mouse model of AD

**DOI:** 10.1186/s13041-024-01092-8

**Published:** 2024-04-30

**Authors:** Hyun-ju Lee, JinHan Nam, Jeong-Woo Hwang, Jin-Hee Park, Yoo Joo Jeong, Ji-Yeong Jang, Su-Jeong Kim, A-Ran Jo, Hyang-Sook Hoe

**Affiliations:** 1 Department of Neural Development and Disease, Korea Brain Research Institute (KBRI), 61, Cheomdan-Ro, Dong-Gu, Daegu, 41068 Korea; 2 Department of Brain & Cognitive Sciences, Daegu Gyeongbuk Institute of Science & Technology (DGIST), Daegu, 42988 Korea

**Keywords:** L-DOPA, Aβ, Tau, Neuroinflammation, Alzheimer’s disease

## Abstract

Dopamine plays important roles in cognitive function and inflammation and therefore is involved in the pathogenesis of neurodegenerative diseases, including Alzheimer's disease (AD). Drugs that increase or maintain dopamine levels in the brain could be a therapeutic strategy for AD. However, the effects of dopamine and its precursor levodopa (L-DOPA) on Aβ/tau pathology in vivo and the underlying molecular mechanisms have not been studied in detail. Here, we investigated whether L-DOPA treatment alters neuroinflammation, Aβ pathology, and tau phosphorylation in 5xFAD mice, a model of AD. We found that L-DOPA administration significantly reduced microgliosis and astrogliosis in 5xFAD mice. In addition, L-DOPA treatment significantly decreased Aβ plaque number by upregulating NEP and ADAM17 levels in 5xFAD mice. However, L-DOPA-treated 5xFAD mice did not exhibit changes in tau hyperphosphorylation or tau kinase levels. These data suggest that L-DOPA alleviates neuroinflammatory responses and Aβ pathology but not tau pathology in this mouse model of AD.

**Supplementary Information:**

The online version contains supplementary material available at 10.1186/s13041-024-01092-8.

## Main text

Alzheimer's disease (AD) is a progressive neurodegenerative disorder characterized by the deposition of amyloid beta (Aβ) plaques and neurofibrillary tangles (NFTs), chronic neuroinflammation, and memory deficits [1]. Dopamine (DA) regulates cognition and neuroinflammation, and in a mouse model of AD, dysfunction of the DA system impairs synaptic plasticity [2]. In addition, DA derivatives or the DA precursor levodopa (L-DOPA) ameliorate Aβ/LPS-induced toxicity and inflammation [3, 4]. However, whether L-DOPA regulates Aβ/tau pathology in vivo and the underlying molecular mechanisms have not been fully investigated.

In the present study, we investigated the effects of L-DOPA on neuroinflammatory responses and Aβ/tau pathology in 5xFAD mice, a model of AD. For this experiment, 4-month-old 5xFAD mice were injected with vehicle (0.9% saline, i.p.) or L-DOPA + benserazide (10 mg/kg and 2.5 mg/kg, respectively, i.p.) daily for 15 days. Then, immunofluorescence staining of brain sections was performed to analyze the therapeutic effects of L-DOPA on gliosis, amyloidopathy, and tau hyperphosphorylation.

**Fig. 1:**
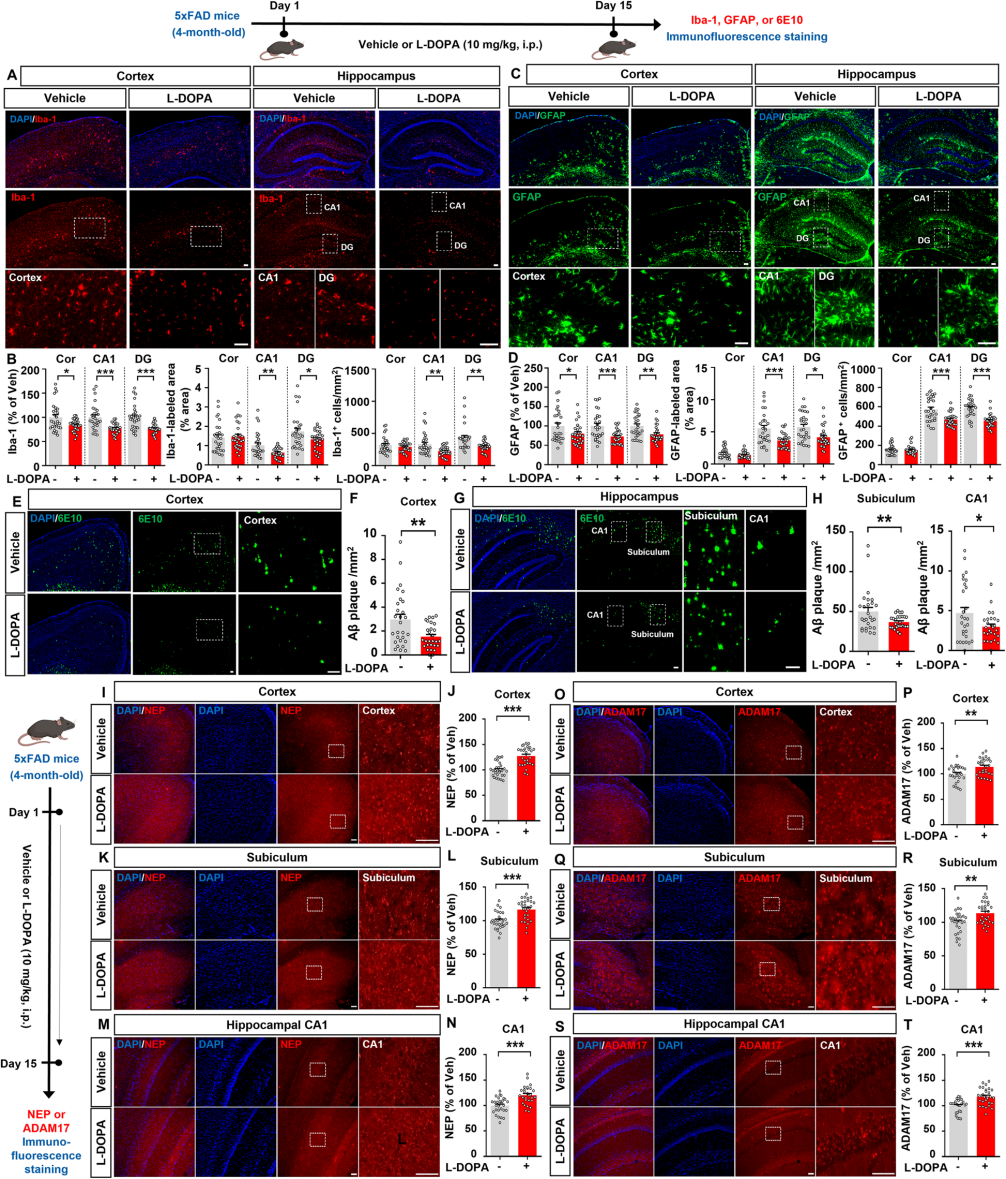
L-DOPA treatment significantly suppresses micro/astrogliosis and Aβ plaque deposition in 5xFAD mice. Four-month-old 5xFAD mice were injected with vehicle (0.9% saline, i.p.) or L-DOPA + benserazide (10 mg/kg and 2.5 mg/kg, respectively, i.p.) daily for 15 days. **A** Immunofluorescence staining of Iba-1. **B** Quantification of data in **A** (*n* = 28 brain slices from 7 mice/group). **C** Immunofluorescence staining of GFAP. **D** Quantification of data in **C** (*n* = 28 brain slices from 7 mice/group). **E**, **G** Immunofluorescence staining of 6E10. **F**, **H** Quantification of data in **E**, **G** (*n* = 27–28 brain slices from 7 mice/group). **I**, **K**, **M** Immunofluorescence staining of NEP. **J**, **L**, **N** Quantification of data in **I**, **K**, **M** (*n* = 27–28 brain slices from 7 mice/group). **O**, **Q**, **S** Immunofluorescence staining of ADAM17. **P**, **R**, **T** Quantification of data in **O**, **Q**, **S** (*n* = 27–28 brain slices from 7 mice/group). *<0.05, **p < 0.01, ***p < 0.001. Scale bar = 100 µm

We found that L-DOPA treatment significantly reduced Iba-1 fluorescence intensity in the cortex and hippocampal CA1 and DG regions in 5xFAD mice compared with vehicle treatment (Fig. 1A, B). In addition, L-DOPA treatment considerably reduced the Iba-1-positive area and the number of Iba-1-positive cells in the hippocampus but not in the cortex (Fig. 1A, B). L-DOPA treatment also significantly diminished GFAP fluorescence intensity in the cortex and hippocampus (Fig. 1C, D). The GFAP-positive area and number of GFAP-positive cells were dramatically reduced in the hippocampal CA1 and DG regions but not in the cortex in L-DOPA-injected 5xFAD mice (Fig. 1C, D). These data suggest that L-DOPA treatment reduces microglial and astrocyte activation in a mouse model of AD.

Sustained activation of glial cells leads to chronic neuroinflammatory responses, which accelerate the accumulation of Aβ plaques and NFTs [5]. Interestingly, DA analogues reduce neuroinflammation by suppressing immune responses [6], and inflammatory responses in microglia decrease DA synthesis in the CNS [7]. Furthermore, imbalanced DA homeostasis in astrocytes induces cognitive impairment [8]. Collectively, these findings and our observation that L-DOPA treatment significantly downregulated microglial and astrocyte activation in 5xFAD mice (Fig. 1C, D) raise the possibility that treatment with the DA precursor L-DOPA increases DA levels in the brain, leading to the attenuation of neuroinflammatory responses. The potential effects of L-DOPA treatment on proinflammatory responses in a mouse model of AD and the molecular mechanisms of action will be addressed in a future study.

Several studies have found that DA regulates Aβ metabolism by preventing Aβ dimerization [9]. Thus, we investigated the effects of L-DOPA treatment on Aβ pathology in a mouse model of AD. We found that treatment with 10 mg/kg L-DOPA significantly decreased Aβ plaque number in the cortex, subiculum, and hippocampal CA1 region in 4-month-old 5xFAD mice (Fig. 1E-H). These data indicate that L-DOPA treatment alleviates β-amyloidosis in a mouse model of AD. In parallel with our findings, DOPAL (a DA metabolite) decreases Aβ oligomerization in human neuroblastoma cells, reducing the cytotoxic effects of Aβ [10]. In addition, in patients with presenile dementia, L-DOPA treatment ameliorates organic brain syndrome and improves visual function with no adverse effects [11]. However, the effects of L-DOPA administration on Aβ deposition remain controversial, as a recent study indicated that L-DOPA treatment (300 nM) increases Aβ production in neuroblastoma cells [12].

Next, we investigated the mechanisms by which L-DOPA mitigates Aβ plaque deposition in 5xFAD mice. First, we analyzed the effect of L-DOPA on the Aβ-degrading enzyme neprilysin (NEP). Compared to vehicle-treated 5xFAD mice, L-DOPA-treated 5xFAD mice exhibited significantly higher NEP levels in the cortex, subiculum, and hippocampal CA1 region, consistent with the reduced Aβ plaque deposition in the same brain regions (Fig. 1I-N). Second, we tested whether L-DOPA attenuates Aβ plaque deposition by modulating the expression of the α-secretase ADAM17 in 5xFAD mice. We found that L-DOPA administration significantly increased ADAM17 expression in the cortex, subiculum, and hippocampal CA1 region in 5xFAD mice (Fig. 1O-T). These results suggest that L-DOPA treatment alleviates Aβ pathology by increasing NEP and ADAM17 levels in this mouse model of AD. Although NEP is a major Aβ-degrading enzyme, it degrades monomeric Aβ and synthetic oligomeric Aβ but not naturally produced Aβ oligomers/fibrils [13]. Therefore, it is possible that L-DOPA treatment suppresses Aβ plaque formation more effectively than Aβ aggregate formation in 5xFAD mice. We will determine whether L-DOPA administration differentially modulates monomers and/or aggregates of Aβ to alter Aβ pathology in a mouse model of AD in a future study.

Since L-DOPA treatment significantly downregulated neuroinflammatory responses and Aβ pathology in a mouse model of AD, we next explored the effects of L-DOPA on tau hyperphosphorylation. L-DOPA treatment did not alter tau phosphorylation at Thr^212^/Ser^214^ (AT100) in 5xFAD mice (Supplementary Fig. 1). In addition, L-DOPA administration did not affect the expression levels of the tau kinases p-GSK3α/β, DYRK1A, and p-CDK5 in 5xFAD mice (Supplementary Figs. 2, 3, and 4). These data suggest that L-DOPA (10 mg/kg, i.p.) treatment does not affect tau hyperphosphorylation or tau kinase levels in a mouse model of AD. However, L-DOPA treatment at higher doses (50 µM in vitro or 100 mg/kg in vivo) significantly upregulates tau phosphorylation in human SH-SY5Y neuroblastoma cells and folate-deficient mice [14]. In addition, the DRD1 agonist SKF38393 increases tau hyperphosphorylation by upregulating tau kinases in vitro [15]. Our findings and those in the literature raise the following question: Why do L-DOPA doses of 100 mg/kg and 10 mg/kg differentially affect tau hyperphosphorylation? The higher dose of L-DOPA might disrupt DA homeostasis, inducing neuroinflammatory responses and ultimately tau pathology. Interestingly, a recent study demonstrated that DOPA decarboxylase (DDC), which converts L-DOPA into DA, is involved in AD pathogenesis. For example, in *C. elegans*, the absence of *bas-1* (which encodes DDC) improves behavioral impairments and suppresses tau phosphorylation by decreasing insoluble tau levels [16], implying a connection between the DA system and tau pathology.

In the present study, injection of 5xFAD mice with L-DOPA (10 mg/kg, i.p.) daily for two weeks did not alter tau pathology. However, this finding may reflect limitations of the AD mouse model and treatment duration, which we will address in future work. First, a longer duration of L-DOPA treatment (> 2 weeks) might have different effects on tau pathology in 5xFAD mice. Second, therapeutic effects of L-DOPA on tauopathy might be observed in human tau transgenic P301S (PS19) mice, another AD model, and we will therefore investigate the effects of L-DOPA on tau pathology (i.e., tau inclusion and propagation of tau aggregates) in human tau transgenic PS19 mice. Third, we used 5xFAD mice to determine the effects of L-DOPA on AD pathology, but 5xFAD mice do not mimic the pathology of AD in humans. To address this limitation, we will brain organoids and/or brain cells derived from induced pluripotent stem cells (iPSCs) from AD patients.

In conclusion, administration of the DA precursor L-DOPA attenuates neuroinflammation in 5xFAD mice. In addition, L-DOPA ameliorates Aβ plaque number in 5xFAD mice by increasing NEP and ADAM17 levels. However, L-DOPA treatment does not affect tau hyperphosphorylation or tau kinase levels in 5xFAD mice. Taken together, our findings suggest that L-DOPA treatment ameliorates neuroinflammatory responses and amyloid plaque deposition but not tau pathology in this mouse model of AD.

### Supplementary Information

**Supplementary Material 1. **

**Supplementary Material 2. **

## Data Availability

All data generated and/or analyzed during this study are included in this published article and its supplementary materials. Materials and methods are presented in the supplementary materials.

## Abbreviations

AD: Alzheimer’s disease
Aβ: Amyloid-β
NFT: Neurofibrillary tangle
NEP: Neprilysin
ADAM17: A disintegrin and metalloproteinase 17
CDK-5: Cyclin-dependent kinase-5
DYRK1A: Dual specificity tyrosine-phosphorylation-regulated kinase 1A

## References

[1] Zhang M, Ganz AB, Hulsman M, Netherlands Brain B, Rozemuller AJM, Scheltens P, Hoozemans JJ, Holstege H (2021). Neuropathological hallmarks of Alzheimer’s disease in centenarians, in the context of aging. Alzheimers Dement.

[2] Nobili A, Latagliata EC, Viscomi MT, Cavallucci V, Cutuli D, Giacovazzo G, Krashia P, Rizzo FR, Marino R, Federici M (2017). Dopamine neuronal loss contributes to memory and reward dysfunction in a model of Alzheimer’s disease. Nat Commun.

[3] Pike AF, Longhena F, Faustini G, van Eik JM, Gombert I, Herrebout MAC, Fayed M, Sandre M, Varanita T, Teunissen CE (2022). Dopamine signaling modulates microglial NLRP3 inflammasome activation: implications for Parkinson's disease. J Neuroinflammation.

[4] Nam E, Derrick JS, Lee S, Kang J, Han J, Lee SJC, Chung SW, Lim MH (2018). Regulatory Activities of Dopamine and Its Derivatives toward Metal-Free and Metal-Induced Amyloid-beta Aggregation, Oxidative Stress, and Inflammation in Alzheimer's Disease. ACS Chem Neurosci.

[5] Heneka MT, Carson MJ, El Khoury J, Landreth GE, Brosseron F, Feinstein DL, Jacobs AH, Wyss-Coray T, Vitorica J, Ransohoff RM (2015). Neuroinflammation in Alzheimer's disease. Lancet Neurol.

[6] Lee JY, Nam JH, Nam Y, Nam HY, Yoon G, Ko E, Kim SB, Bautista MR, Capule CC, Koyanagi T (2018). The small molecule CA140 inhibits the neuroinflammatory response in wild-type mice and a mouse model of AD. J Neuroinflammation.

[7] Vidal PM, Pacheco R (2020). The Cross-Talk Between the Dopaminergic and the Immune System Involved in Schizophrenia. Frontiers in Pharmacology.

[8] Petrelli F, Dallérac G, Pucci L, Calì C, Zehnder T, Sultan S, Lecca S, Chicca A, Ivanov A, Asensio CS (2020). Dysfunction of homeostatic control of dopamine by astrocytes in the developing prefrontal cortex leads to cognitive impairments. Mol Psychiatry.

[9] Li J, Zhu M, Manning-Bog AB, Di Monte DA, Fink AL (2004). Dopamine and L-dopa disaggregate amyloid fibrils: implications for Parkinson's and Alzheimer's disease. FASEB J.

[10] Cataldi R, Chia S, Pisani K, Ruggeri FS, Xu CK, Šneideris T, Perni M, Sarwat S, Joshi P, Kumita JR (2021). A dopamine metabolite stabilizes neurotoxic amyloid-β oligomers. Communications Biology.

[11] Jellinger K, Flament H, Riederer P, Schmid H, Ambrozi L (1980). Levodopa in the treatment of (PRE) senile dementia. Mech Ageing Dev.

[12] Lu J, Li X, Wang Q, Pei G (2017). Dopamine D2 receptor and β-arrestin 2 mediate Amyloid-β elevation induced by anti-parkinson's disease drugs, levodopa and piribedil, in neuronal cells. PLoS ONE.

[13] Grimm MO, Mett J, Stahlmann CP, Haupenthal VJ, Zimmer VC, Hartmann T (2013). Neprilysin and Abeta Clearance: Impact of the APP Intracellular Domain in NEP Regulation and Implications in Alzheimer's Disease. Front Aging Neurosci.

[14] Teodoro B, Erland A, Brandi W, Viyada N-C, Jean-Marie S, Estelle S (2012). Acute Administration of L-Dopa Induces Changes in Methylation Metabolites, Reduced Protein Phosphatase 2A Methylation, and Hyperphosphorylation of Tau Protein in Mouse Brain. J Neurosci.

[15] Lebel M, Patenaude C, Allyson J, Massicotte G, Cyr M (2009). Dopamine D1 receptor activation induces tau phosphorylation via cdk5 and GSK3 signaling pathways. Neuropharmacology.

[16] Kow RL, Sikkema C, Wheeler JM, Wilkinson CW, Kraemer BC (2018). DOPA Decarboxylase Modulates Tau Toxicity. Biol Psychiatry.

