# Supplementary material for: L-DOPA regulates neuroinflammation and Aβ pathology through NEP and ADAM17 in a mouse model of AD
Source: Mol Brain. 2024 Apr 30;17:21. doi: 10.1186/s13041-024-01092-8 (PMC11059733; doi:10.1186/s13041-024-01092-8)
Supplement: Supplementary file 1 — Supplementary Material 1. [file 13041_2024_1092_MOESM1_ESM.docx]

**L-DOPA regulates neuroinflammation and Aβ pathology through NEP and ADAM17 in a mouse model of** **AD**

**Hyun-ju Lee^1,3^, Jin Han Nam^1,3^, Jin-Hee Park^1,2^, Jeong-Woo Hwang^1^, Yoo Joo Jeong^1,2^, Ji-Yeong Jang^1,2^, Su-Jeong Kim^1^, A-Ran Jo^1^, Hyang-Sook Hoe^1, 2,*^**

^1^Department of Neural Development and Disease, Korea Brain Research Institute (KBRI), 61, Cheomdan-ro, Dong-gu, Daegu, Korea 41068; ^2^Department of Brain & Cognitive Sciences, Daegu Gyeongbuk Institute of Science & Technology (DGIST), Daegu, Korea, 42988; ^3^These authors contributed equally to this work.

**Corresponding author:**

***Hyang-Sook Hoe, PhD;** Department of Neural Development and Disease. Korea Brain Research Institute (KBRI); 61 Cheomdan-ro, Dong-gu, Daegu, Korea, 41068; E-mail: [sookhoe72@kbri.re.kr](mailto:sookhoe72@kbri.re.kr)

**
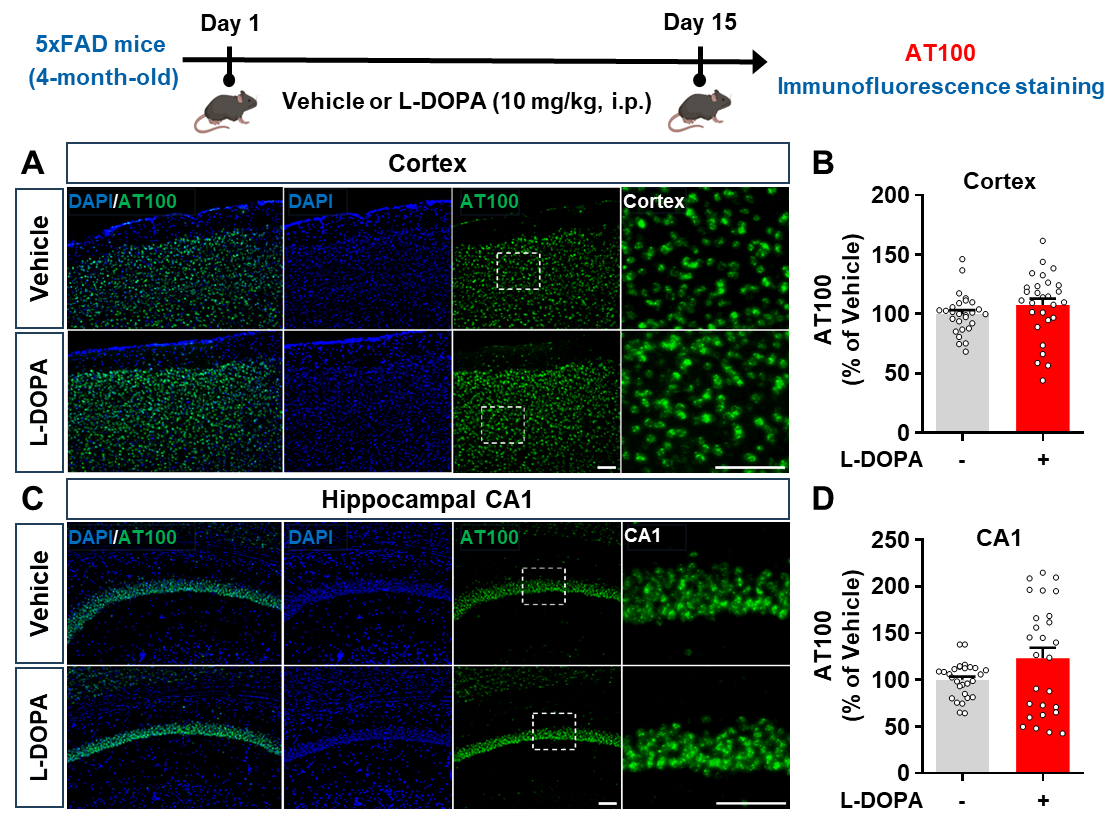
Supplementary Fig 1. L-DOPA treatment does not alter tau hyperphosphorylation in 5xFAD mice**. **A, C** Immunofluorescence staining of AT100 in 5xFAD mice injected with vehicle (0.9% saline, i.p.) or L-DOPA + benserazide (10 mg/kg and 2.5 mg/kg, respectively, i.p.) daily for 15 days. **B, D** Quantification of data from A and C, respectively (n = 27-28 brain slices from 7 mice/group). Scale bar = 100 µm.

**
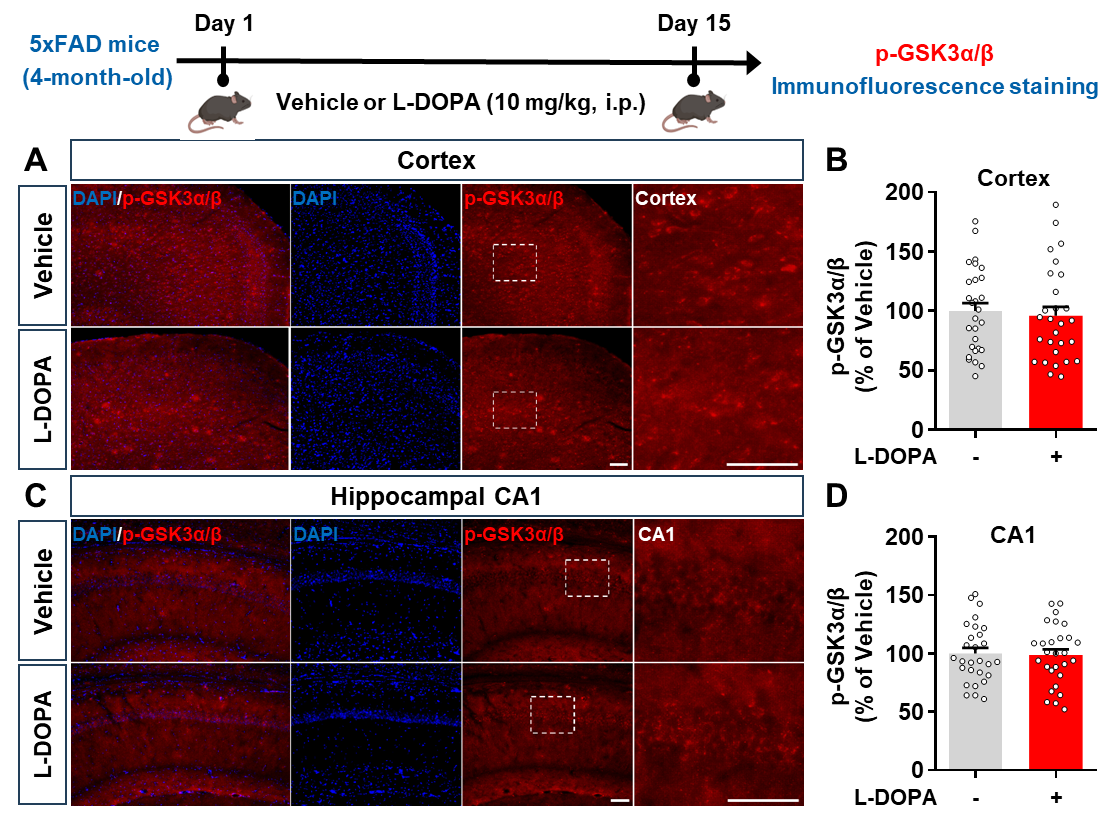
Supplementary Fig 2. L-DOPA administration does not affect tau kinase p-GSK3α/β in 5xFAD mice.** **A, C** Immunofluorescence staining of p-GSK3α/β in 5xFAD mice injected with vehicle (0.9% saline, i.p.) or L-DOPA + benserazide (10 mg/kg and 2.5 mg/kg, respectively, i.p.) daily for 15 days. **B, D** Quantification of data from A and C, respectively (n = 28 brain slices from 7 mice/group). Scale bar = 100 µm.

**
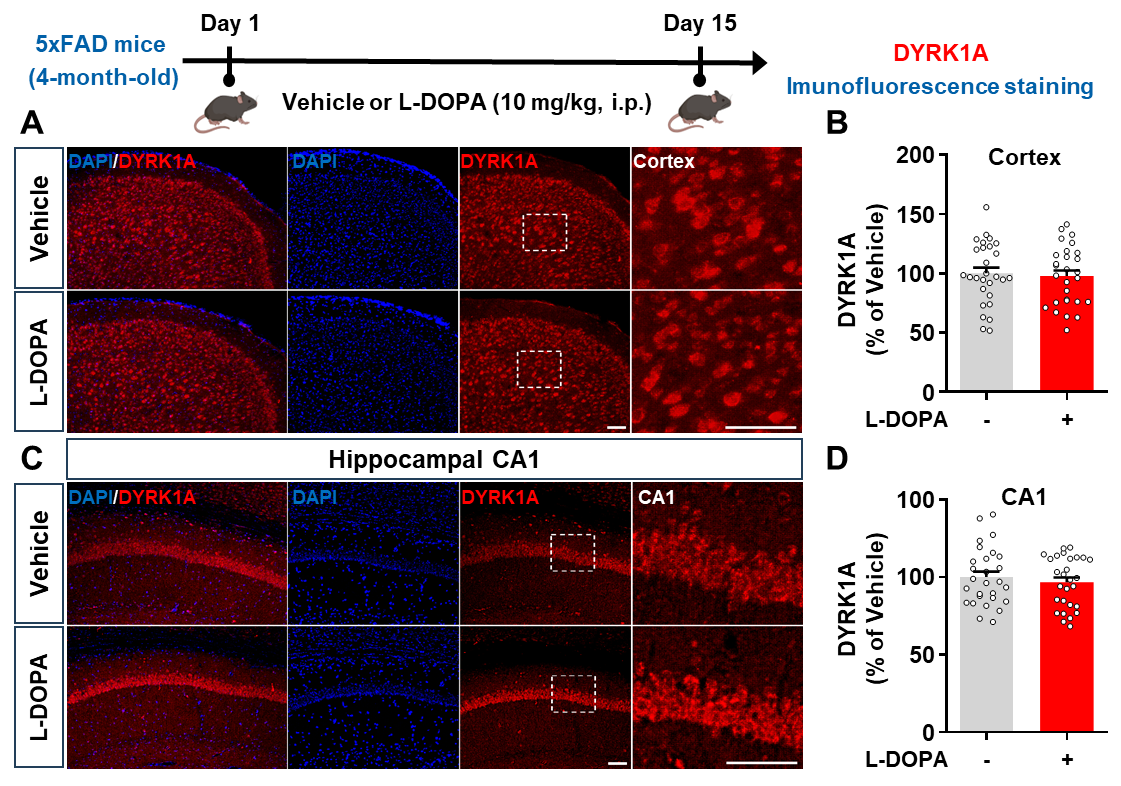
Supplementary Fig 3. L-DOPA does not reduce tau kinase DYRK1A levels in 5xFAD mice.** **A, C** Immunofluorescence staining of DYRK1A in 5xFAD mice injected with vehicle (0.9% saline, i.p.) or L-DOPA + benserazide (10 mg/kg and 2.5 mg/kg, respectively, i.p.) daily for 15 days. **B, D** Quantification of data from A and C, respectively (n = 28 brain slices from 7 mice/group). Scale bar = 100 µm.


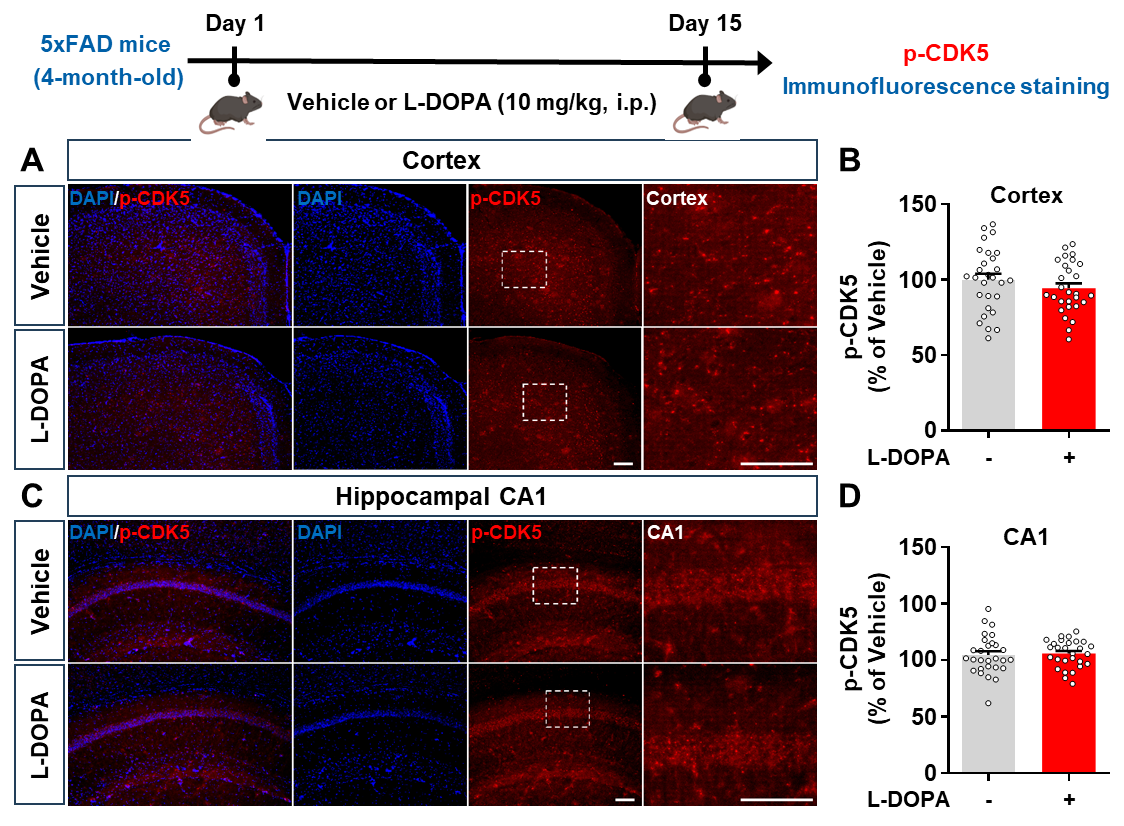


**Supplementary Fig 4**. L-DOPA treatment does not affect tau kinase p-CDK5 levels in 5xFAD mice. **A, C** Immunofluorescence staining of p-CDK5 in 5xFAD mice injected with vehicle (0.9% saline, i.p.) or L-DOPA + benserazide (10 mg/kg and 2.5 mg/kg, respectively, i.p.) daily for 15 days. **B, D** Quantification of data from A and C, respectively (n = 28 brain slices from 7 mice/group). Scale bar = 100 µm.

**Materials and methods**

**Levodopa (L-DOPA)**

L-DOPA (Cat. No. PHR1271) and benserazide hydrochloride (Cat. No. B7283) were purchased from Sigma–Aldrich (St. Louis, MO, USA). Benserazide hydrochloride is a dopamine decarboxylase inhibitor that is administered with L-DOPA in PD to prevent conversion L-DOPA to dopamine[1]. Thus, L-DOPA (10 mg/kg) and benserazide hydrochloride (2.5 mg/kg) were dissolved together in 0.9% saline and administered intraperitoneally (i.p.) in vivo.

**5xFAD mice**

To investigate the effect of L-DOPA on AD pathology, including Aβ, tau, and neuroinflammatory responses, 5xFAD transgenic male mice (MMRRC Stock 34848; B6.Cg-Tg (APPSwFlLon,PSEN1*M146L*L286V)6799Vas/Mmjax) were purchased from Jackson Laboratory (Bar Harbor, ME, USA). This AD mouse model carries a transgene overexpressing two mutations in human presenilin 1 (PSEN1 M146L, L286V) under the control of the Thy1 promoter and a transgene overexpressing three mutations in human APP (Swedish mutation K670N, M671L; Florida mutation I716V; London mutation V717I). These five mutations are associated with familial AD. Genotyping of the human APP and PSEN genes was conducted by RT–PCR analysis of tail genomic DNA. All experimental procedures were approved by the institutional biosafety committee (IBC) and performed in accordance with approved animal protocols of the Korea Brain Research Institute (KBRI, approval no. IACUC-22-00045). This study is reported in accordance with ARRIVE guidelines.

**Study design**

The mice were housed 3 or 4 mice per cage and maintained in a pathogen-free facility with a 12-h light/dark cycle and a temperature of 22 °C. To investigate the effects of L-DOPA on AD pathology, 4-month-old 5xFAD mice were assigned randomly into two groups and injected with a mixture of L-DOPA and benserazide hydrochloride (10 mg/kg and 2.5 mg/kg, respectively, i.p.) or vehicle (0.9% saline, i.p.) daily for 15 days (n = 7/group). All mice were anesthetized by 2,2,2-Tribromoethanol (Sigma Aldrich, St. Louis, MO, USA, 2.5 %, v/v, 150 mg/kg, intraperitoneal injection) 2 h after the final injection. Subsequently, 5xFAD mice were perfused and fixed with PBS and 4% paraformaldehyde respectively, and post-fixed overnight in 4% paraformaldehyde at 4 °C and subsequently immersed in 30% sucrose solution for 3 days for dehydration. The brain slices were sectioned into 30-µm-thick coronal slices using a cryostat (Leica CM1850, Wetzlar, Germany) and immunofluorescence staining was conducted.

**Immunofluorescence staining**

To perform immunofluorescence staining, the brain slices were permeabilized with PBS containing 0.2% Triton X-100 (PBST) and incubated with blocking solution [10% normal goat serum (Vector Laboratories, S-1000-20, Burlingame, CA) in PBST] for 2 h at room temperature. Next, immunofluorescence staining was conducted with anti-Iba-1, anti-GFAP, anti-6E10, anti-NEP, anti-ADAM17, anti-AT100 (to detect tau phosphorylation at Thr^212^/Ser^214^), anti-p-GSK3α/β, anti-DYRK1A, and anti-p-CDK5 antibodies diluted in PBST for 24–72 h at 4 °C. After washing 3 times with PBST, the brain sections were incubated with the appropriate goat anti-rabbit, mouse, or rat secondary antibody conjugated with Alexa Fluor 488 or 594 for 2 h at room temperature. Finally, the slices were washed with PBST, PBST/DAPI, and PBS and mounted on glass slides with antifade mounting solution containing DAPI (Vector Laboratories, H-1200-10, Burlingame, CA). Brain slices from each group (5xFAD mice treated with vehicle or L-DOPA + benesrazide) were randomly selected for immunofluorescence staining to remove the non-biological variation and batch effect. The immunofluorescence staining images were acquired by fluorescence microscopy (DMi8, Leica Microsystems, Wetzlar, Germany). The immunofluorescence staining was performed with same reagents, the equivalent experimental process, the same antibodies, and the images were captured with identical conditions. Detailed information on the primary and secondary antibodies is provided in supplementary Table 1.

**Supplementary table 1.** Detailed information on the antibodies used in this study.

| **Primary antibodies** | | | | | | | |
| --- | --- | --- | --- | --- | --- | --- | --- |
| **Immunogen** | **Host Species** | | **Manufacturer** | | **Catalog No.** | | **Dilution** |
| Iba-1 | Rabbit | | Wako | | 019-19741 | | 1:500 |
| GFAP | Rat | | Invitrogen | | 13-0300 | | 1:500 |
| 6E10 | Mouse | | BioLegend | | 803002 | | 1:500 |
| NEP | Rabbit | | Millipore | | Ab5458 | | 1:200 |
| ADAM17 | Rabbit | | Abcam | | Ab2051 | | 1:200 |
| AT100 | Mouse | | Invitrogen | | Mn1060 | | 1:200 |
| p-GSK3α/β | Rabbit | | Abcam | | Ab75745 | | 1:200 |
| DYRK1A | Rabbit | | Abcam | | Ab180910 | | 1:200 |
| p-CDK5 | Rabbit | | LSBio | | LS-C354604-100 | | 1:200 |
| **Secondary antibodies** | | | | | | | |
| **Antibody** | | **Manufacturer** | | **Catalog No.** | | **Dilution** | |
| Goat-anti-mouse IgG, 488 | | Invitrogen | | A11001 | | 1:200 | |
| Goat-anti-rat IgG, 488 | | Invitrogen | | A11006 | | 1:200 | |
| Goat-anti-rabbit IgG, 594 | | Invitrogen | | A11012 | | 1:200 | |

**Quantification of immunofluorescence staining**

To evaluate the effects of L-DOPA treatment on microglial and astroglial activation, we measured Iba-1/GFAP fluorescence intensity, Iba-1/GFAP labeled area, and Iba-1/GFAP positive cells. Specifically, the area of brain region was quantified a region of interest (ROI) in a DAPI fluorescence using Image J software (US National Institutes of Health, Bethesda, MD, USA). The selected ROIs were overlaid on matching with red or green fluorescence images, and the fluorescence intensity, labeled area, and positive cells were measured. The parameters were calculated as follows: [Fluorescence intensity (% of vehicle) = fluorescence intensity/ROI/average of vehicle group*100]; [Labeled area (% of area) = percent Iba-1 or GFAP labeled area/ROI]; [Positive cells (cells/mm^2^) = number of Iba-1 or GFAP positive cells/ROI].

To determine whether L-DOPA alters NEP, IDE, ADAM10, ADAM17, AT100, p-GSK3α/β, DYRK1A, or p-CDK5 levels in the brain, the area of brain region was quantified a region of interest (ROI) in a DAPI fluorescence using Image J software. The selected ROIs were overlaid on matching with red or green fluorescence images, and the fluorescence intensity was measured as describe above.

To determine the effects of L-DOPA on Aβ plaque number, the number of Aβ plaques were counted according to the previously reported with minor modifications [2, 3]. Briefly, quantification of 6E10-positive Aβ plaques numbers was obtained in green fluorescence images by using Particle Analysis plugin of ImageJ with custom thresholding (with a diameter 10 µm or more). The area of brain region was quantified a region of interest (ROI) in a DAPI fluorescence using Image J software. To assess the amyloid burden per mm^2^, the total cell numbers of 6E10-positive Aβ plaques were divided by the area of manually drawn ROIs in cortex or hippocampus.

All data were performed with Grubbs’ test and the most extreme value was excluded (Significant outlier, *p* < 0.05). In addition, quantification of immunofluorescence staining was conducted by 2 independent researcher who did not participate in this study.

**Statistical analysis**

Statistical analysis and graph illustration were performed using GraphPad Prism 8 (GraphPad Software, San Diego, CA, USA). Data are presented as individual data points and the mean ± SEM. The unpaired two-tailed *t*-test was used for comparisons between two groups. Asterisks indicate significance (* *p* < 0.05, ***p* < 0.01, and *** *p* < 0.001).

**References**

1. Shimozawa A, Fujita Y, Kondo H, Takimoto Y, Terada M, Sanagi M, Hisanaga S-i, Hasegawa M: **Effect of L-DOPA/Benserazide on Propagation of Pathological α-Synuclein**. *Frontiers in Neuroscience* 2019, **13**.

2. Zhiqiang L, Carlo C, Aaron S, Roa H, Jaime G: **CX3CR1 in Microglia Regulates Brain Amyloid Deposition through Selective Protofibrillar Amyloid-β Phagocytosis**. *The Journal of Neuroscience* 2010, **30**(50):17091.

3. Leinenga G, Götz J: **Scanning ultrasound removes amyloid-β and restores memory in an Alzheimer’s disease mouse model**. *Science Translational Medicine* 2015, **7**(278):278ra233-278ra233.
